# An Observational Study of the Impact of a Computerized Physician Order Entry System on the Rate of Medication Errors in an Orthopaedic Surgery Unit

**DOI:** 10.1371/journal.pone.0134101

**Published:** 2015-07-24

**Authors:** Fabien Hernandez, Elyes Majoul, Carlota Montes-Palacios, Marie Antignac, Bertrand Cherrier, Levon Doursounian, Jean-Marc Feron, Cyrille Robert, Gilles Hejblum, Christine Fernandez, Patrick Hindlet

**Affiliations:** 1 AP–HP, Saint Antoine Hospital, Pharmacy Department, Paris, France; 2 AP–HP, Saint Antoine Hospital, Orthopaedic Surgery Department, Paris, France; 3 AP-HP, Saint Antoine Hospital, Anaesthetics and Intensive Care Department, Paris, France; 4 Sorbonne Universités, UPMC Univ Paris 06, INSERM, Institut Pierre Louis d’Epidémiologie et de Santé Publique (IPLESP UMRS 1136), Paris, France; 5 Univ Paris-Sud, Faculty of Pharmacy, Chatenay-Malabry, France; York University, CANADA

## Abstract

**Aim:**

To assess the impact of the implementation of a Computerized Physician Order Entry (CPOE) associated with a pharmaceutical checking of medication orders on medication errors in the 3 stages of drug management (i.e. prescription, dispensing and administration) in an orthopaedic surgery unit.

**Methods:**

A before-after observational study was conducted in the 66-bed orthopaedic surgery unit of a teaching hospital (700 beds) in Paris France. Direct disguised observation was used to detect errors in prescription, dispensing and administration of drugs, before and after the introduction of computerized prescriptions. Compliance between dispensing and administration on the one hand and the medical prescription on the other hand was studied. The frequencies and types of errors in prescribing, dispensing and administration were investigated.

**Results:**

During the pre and post-CPOE period (two days for each period) 111 and 86 patients were observed, respectively, with corresponding 1,593 and 1,388 prescribed drugs. The use of electronic prescribing led to a significant 92% decrease in prescribing errors (479/1593 prescribed drugs (30.1%) vs 33/1388 (2.4%), p < 0.0001) and to a 17.5% significant decrease in administration errors (209/1222 opportunities (17.1%) vs 200/1413 (14.2%), p < 0.05). No significant difference was found in regards to dispensing errors (430/1219 opportunities (35.3%) vs 449/1407 (31.9%), p = 0.07).

**Conclusion:**

The use of CPOE and a pharmacist checking medication orders in an orthopaedic surgery unit reduced the incidence of medication errors in the prescribing and administration stages. The study results suggest that CPOE is a convenient system for improving the quality and safety of drug management.

## Introduction

In 1999, the Institute of Medicine estimated that 44,000 to 98,000 people die annually in United States hospitals because of medical errors. Medication errors in hospitals are common, expensive, sometimes harmful to the patient [1, 2] and are responsible for 7,000 deaths per year in the United States.[3] According to a recent study, adverse drug events occurring in hospital cost more than $ 3,000 on average and increase the length of stay by 3.1 days.[4]

Since the 1960s, medication errors have been the subject of many studies and the incidence has differed greatly according to the methodology used and the country and healthcare system under study. Moreover, the methods used to assess medication errors are heterogeneous thus making comparison between studies hazardous.[5] Medication errors can occur in all stages of the medication process, from prescribing to dispensing and administration of drugs.[6]

Medical prescriptions can lead to medication errors due to the following factors: insufficient knowledge of drugs and their use in the context of a patient's condition, non-compliance with good practices, nomenclature factors (ambiguous prescription, incorrect drug name, dosage form, or abbreviation).[7]

Several risk factors associated with administration errors have been reported. For instance, Westbrook et al. showed that nurses’ interruptions increase the risk of administration error including the risk of serious error.[8] The risk of administration error also appears to be higher for drugs administered intravenously.[9, 10]

Among the various tools which have been proposed for reducing the number of medication errors, the use of a Computerized Physician Order Entry (CPOE) system is recommended by several European and American institutions.[11, 12] CPOE software allows physicians to enter medical orders by computer. CPOE systems often include functionalities such as drug dosage support, alerts about harmful interactions and clinical decision support, which may further reduce errors. CPOE mainly reduces errors associated with hand-written prescriptions by reducing the number of illegible orders, incomplete orders or incorrect dosages.[13–16] A systematic review [17] indicates that 23 of the 25 selected studies reported a 13–99% decrease in the relative risk of medication errors after the implementation of CPOE, while one study found no change [18] and one study resulted in a significant increase in the relative risk of medication errors.[19] Other studies have reported the introduction of new errors or an increase in mortality related to implementation.[20, 21] Interestingly, Bates et al showed that the reduction of medication errors induced by a basic CPOE system is further enhanced after the implementation of additionnal decision support features [22]. In the end, Radley et al [23] advocate that the global positive impact of CPOE should be more widespread in hospitals.

Studies investigating the impact of the use of CPOE on medication errors occurring at the different stages of medication delivery, i.e., prescription, dispensing and administration, are fewer. Bates et al. showed a significant decrease in the frequency of non-intercepted serious medication errors at the stages of dispensing and administration after the implementation of CPOE.[24] However, the distribution of the types of errors occurring at each of these two stages was not reported, impairing a clear understanding of the mechanism by which CPOE reduces the proportion of medication errors.

The impact of the use of CPOE in an orthopaedic surgery unit has not been previously evaluated. Nevertheless medication errors are common in an orthopaedic surgery ward.[25] In this study, the authors stated that surgeons are unfamiliar with chronic treatment. In our study, we propose to observe the impact of the implementation of CPOE on prescribing, dispensing and administration of drugs in an orthopaedic surgery unit.

## Methods

### Study design

A before and after observational study was designed to observe the impact of the implementation of a CPOE system on prescription, dispensing and administration of drugs.

### Setting

The study was conducted in the 66-bed orthopaedic surgery ward of a 700-bed teaching hospital in Paris, France. The department was divided into 3 wards and the average length of stay was 6.4 days (2012). The medication process was the following: first, the order was made by physicians (using handwritten orders before software implementation and CPOE thereafter); second, according to these orders, the nursing staff dispensed drugs for each patient on trolleys devoted to medicine dispensation: each trolley contains 12 individual pill dispensers, one for each patient who is identified by name and room number. Each pill dispenser is planned for a 24-hour period, and is accordingly composed of three inner compartments corresponding to the three drug rounds (i.e. 8am, 12am, and 6pm). Medications were dispensed in the pill dispensers from a stock available in the satellite ward pharmacy (except pharmaceutical controlled drugs, such as antibiotics or expensive drugs requiring the validation by a pharmacist before dispensing). Finally, the nursing staff administered the drugs during their drug rounds.

Prior to CPOE implementation, all units used paper medication charts for physicians’ handwritten orders. These charts were then used by nurses to dispense drugs in the pill dispensers and as medication administration charts, thus no retranscription was required. Administration (drug name, dose, time at administration, nurse’s signature) was directly recorded on medication administration charts, in an allocated space on the order sheets. After implementation of the CPOE, the system included an electronic medication administration record. The order was available for nurses on a laptop computer attached to the medicine dispensing trolley. The administration was electronically recorded (drug name, dose, time at administration, nurse’s electronic signature) by the nurse after administration to the patient.

After implementation of the CPOE system, all orders were routinely checked by a pharmacist (it was not the case prior to CPOE implementation). Medications placed into the pill dispenser were not checked by the pharmacist before or after CPOE.

Prescriptions were handwritten until May 14^th^ 2013 (implementation of the CPOE system). The study was based on a “before-after” design (pre- and post- implementation study observation periods). In order to estimate the appropriate duration of data collection (period durations), we conducted a pilot study which determined that a two-day observation period was likely to result in 1000 drug prescriptions. This number of prescriptions was judged sufficient for detecting common medication errors. Therefore the study pre-implementation observation period took place during two 24-hour periods (Jan 15–16^th^ 2013 and Feb 28^th^-Mar 1^st^ 2013) as well as the study post-implementation observation period (Jun 17–18^th^ 2013 and Jul 3–4^th^ 2013).

The observer team was composed of six pharmacists (two senior, two residents, and two students) who had been previously trained in data collection. They observed dispensing and administration processes and performed this task blind to the contents of the corresponding prescriptions.

Six nurses simultaneously administrated drugs within the 3 wards (2 nurses per ward). Each observer was assigned to a nurse and shadowing was done simultaneously in all wards. Every day, the pharmacists observed the nurses during the three medication rounds (i.e. 8 am, 12 am, 6 pm) and collected data on the dispensing and administration of all medications delivered to patients, while, at the end of the day, corresponding initial prescription charts were collected. Whenever a pharmacist was aware of a potential serious error at the dispensing or administration phase, he(she) intervened to prevent it but such an event was nevertheless registered as an error. In addition, nurses were asked if any medication had been administered since the previous medication round, and if any drug present in the prescription chart had not been administered and the reason why.

### CPOE system

From May 14^th^ 2013, commercial software was used throughout the orthopaedic surgery unit. Physicians and residents were required to enter their own orders in the system in the post-CPOE period. In this system, the prescription is entered through a drug selection menu. Dosage, route of administration and frequency are mandatory for prescription validation, and the system displays alert messages if any of these fields are not specified. Free-text fields are available for extra information. The software also includes alerts for drug-allergy checking, therapeutic duplications, dose-range and age-based checking, and drug-drug interactions. None of the alerts prevents the prescriber from continuing with the order. The prescriber may also use predefined protocols such as analgesic care or prophylactic antibiotic treatment sets.

### Participants

All physicians and nurses present in the department during the observation periods were included in the study.

### Variables

#### Prescription stage

To assess the impact of the CPOE implementation on prescriptions, we first studied the procedural compliance with the medication order (procedural errors). A medication order was considered non-compliant whenever patient identification was incomplete (name, gender and date of birth), prescriber identification was incomplete (name or signature), or whenever the date of the prescription was missing. We then focused on errors having potential impact on patients (prescribing errors): unclear prescription (i.e. illegible, ambiguous or use of abbreviations), no dosage or an incorrect dosage (that does not exist or cannot be easily obtained from the current forms commercially available), lack of administration route stated (if the drug was available for several routes), duplicated therapy. The impact of CPOE on prescribing was analyzed by comparing the proportion of errors (number of errors/number of drugs prescribed) before and after the implementation.

#### Dispensing and administration stage

Errors in these two stages were defined as dispensing or administration not compliant with the prescription. We focused on the following errors: incorrect dose, incorrect time, wrong patient, drug omission or unordered drug. An incorrect time was defined as the dispensing or administration of a drug with a delay exceeding 1 hour before or after the time scheduled on the prescription. Drug omission was defined as the absence of dispensing or administration of a drug without justification by the nurse. Finally, a drug was considered as unordered if it was dispensed or administered but not prescribed by the doctor.

To assess the impact of CPOE on drug dispensing and administration, we only compared the drugs that were correctly prescribed pre-CPOE and post-CPOE (i.e. clear prescription with route of administration and dosage). Dispensing and administration from incorrect prescriptions were not included in the analysis as it was not possible to determine if dispensing and administration were in accordance with the prescription.

The error rate was calculated using the Total Opportunities for Errors (TOE), which is the sum of all doses ordered plus all unordered doses.[26] The drug dispensing error rate and the drug administration error rate were then calculated as the number of dispensing and administrations with one or more errors, respectively, divided by the corresponding TOE and multiplied by 100.

### Data sources

During the study, the following patient characteristics were collected from patient files: age, gender, date of hospitalization, cause of hospitalization and type of hospitalization (scheduled or unscheduled). During the observation of drug dispensing from the medicine dispensing trolleys, the following data were collected: drug name, dosage, dosage form, quantity and time scheduled for administration. During drug administration, the following data were collected: drug name, dosage, quantity, route of administration and time. If a drug administered was not already dispensed in the patient pill dispenser, the observer stated the cause of non-dispensing (extemporaneous preparation, opioids, dispensing omission etc.). After the three drug rounds, the following data were collected from prescription charts: patient identification (age, gender, date of birth), prescriber’s identification (name and signature), prescription date, drug name, dosage, route of administration, dosage form, frequency of administration and condition of administration (prn drugs). All data collected at the dispensing and administration stages were compared to the prescription, which was the reference in our study. These data were collected on an observation form by a single observer. The form was tested during the pilot study.

This study is a clinical audit conducted as part of an evaluation of professional practices (i.e. prescribing, dispensing and administration of drugs in our orthopaedic surgery unit). This type of evaluation implied the observation of these three stages of the medication process. All data were collected anonymously, no approval of an ethics committee was therefore necessary, and no written consent or permission to use the data was submitted to the patients. The hospital granted permission for this study to be performed. The data collection was declared to the National Commission of Information Technology and Liberties.

### Bias

In order to lower bias, a disguised direct observation was used for the detection of errors in the dispensing and the administration of drugs as described earlier.[27] Disguised direct observation means that pharmacists observed nurses who performed the dispensing and administration of drugs. These nurses were not aware of the aim of the study. Additionally, nurses and physicians were unaware of the day of the survey. Finally, the observers were blind to the contents of the prescription.

### Quantitative variables and statistical methods

Qualitative variables were reported using frequencies (percentages) and comparisons on such variables were made with the chi-square test or the Fisher's exact test. Quantitative variables were reported as median [interquartile range, IQR] and comparisons on such variables were made with the non-parametric Mann-Whitney test. All reported tests were two-tailed, and P ≤ 0.05 was predetermined to represent statistical significance. All statistical analyses were carried out with GraphPad Prism version 5 (GraphPad Software, Inc., La Jola, CA, USA).

## Results

### Patients and Staff

In the pre-CPOE period, 111 patients were included in the study, with 1,593 corresponding prescribed medications in 306 medication orders (one medication order corresponding to one round). The median age was 64 years [48; 81]. The population comprised 54.0% women and hospitalizations were scheduled for 45.9% of patients. The median length of stay between the beginning of the hospitalization and the day of the survey was 5 days [2; 12] and the median number of drugs prescribed per patient was 8 drugs [6; 11]. The staff involved in the pre-CPOE period included the daily presence of 4 physicians, 6 residents, and 12 to 14 nurses.

In the post-CPOE period, 86 patients were included in the study with 1,388 corresponding prescribed medications in 258 medication orders. The median age was 61 years [44; 83]. The population comprised 55.8% women and hospitalizations were scheduled for 36.0% of patients, the median length of stay between the beginning of the hospitalization and the day of the survey was 4 days [1; 11] and the median number of drugs prescribed per patient was 9 drugs [6; 13]. The staff involved in the post-CPOE period included the daily presence of 4 physicians, 6 residents, and 12 to 13 nurses. No significant difference was found in patient characteristics between the pre-CPOE period and the post-CPOE period (Table 1). There was no missing data for any participant.

**Table 1:** Patients’ characteristics before-after CPOE implementation.

Variable	Pre-CPOE (N = 111)	Post-CPOE (N = 86)	*p* Value
**Age (years)**	64 [48; 81]	61 [44; 83]	0.340
**Women (%)**	54.0	55.8	0.885
**Scheduled hospitalization / Unscheduled hospitalization (%)**	45.9/53.1	36.0/64.0	0.190
**Length of stay (days)**	5 [2; 12]	4 [1; 11]	0.212
**Number of drugs/patient**	8 [6; 11]	9 [6; 13]	0.078

### Prescription stage

#### Procedural errors

In the pre-CPOE period, procedural errors were observed in 35.6% of prescriptions. There were 64 prescriptions out of 306 (20.9%) in which prescriber identification was incomplete, 40 (13.1%) in which patient identification was incomplete, and 5 (1.6%) in which the date was missing. After CPOE implementation, no procedural errors were detected.

#### Prescribing errors

The CPOE implementation resulted in a 92% decrease in prescribing errors (Table 2): there were 479 medications with a prescribing error out of 1593 (30.1%) in the pre-CPOE period while there were 33 medications with a prescribing error out of 1388 (2.4%) in the post-CPOE period (p<0.0001). The most common errors observed during the pre-CPOE period (unclear prescription, route omission, dosage omission) were totally eliminated during the CPOE period. The rate of duplicated therapy observed during the pre-CPOE period (0.4%) substantially increased (1.8%) during the post-CPOE period.

**Table 2:** Prescribing errors before and after CPOE implementation.

Type of error	Pre-CPOE (N = 1593)*	Post-CPOE (N = 1388)*	p Value
**Total (n, %)**	479 (30.1)	33 (2.4)	< 0.0001
**Unclear (n, %)**	250 (15.7)	0 (0)	
**Omission of route (n, %)**	135 (8.5)	0 (0)	
**Omission of dosage (n, %)**	48 (3.0)	0 (0)	
**Incorrect dosage (n, %)**	39 (2.4)	8 (0.6)	
**Duplicated therapy (n, %)**	7 (0.4)	25 (1.8)	

* Total number of drugs prescribed

### Dispensing stage

The TOE was 1,219 medications (1,161 correctly prescribed + 58 unordered drugs) in the pre-CPOE period and 1,407 medications (1,388 + 19) after the introduction of computerized prescribing (Table 3). The error rate at the stage of drug dispensing observed in the pre- and post-CPOE period was not significantly different, 35.3% (n = 430) and 31.9% (n = 449), respectively (p = 0.07). The most frequent error was the omission of dispensing, followed by the dispensing of a wrong dosage, the dispensing of unordered drugs, and then the dispensing at the incorrect time. Analgesics and laxatives accounted for 50% of drug dose omissions both before and after CPOE implementation. Finally, the CPOE implementation resulted in a 70% decrease in the dispensing rate of unordered drugs (4.8% pre-CPOE versus 1.3% post-CPOE).

**Table 3:** Dispensing errors before and after CPOE implementation.

Type of error	Pre-CPOE (N = 1219)*	Post-CPOE (N = 1407)*	p Value
**Total (n, %)**	430 (35.3)	449 (31.9)	0.07
**Omission (n, %)**	288 (23.6)	356 (25.3)	
**Incorrect dosage (n, %)**	58 (4.8)	53 (3.8)	
**Wrong time (n, %)**	26 (2.1)	21 (1.5)	
**Unordered drug (n, %)**	28 (4.8)	19 (1.3)	

*Number of total opportunities for error

### Administration stage

The TOE amounted to 1,222 medications (1,161 correctly prescribed + 61 unordered drugs) in pre-CPOE period and 1,407 (1,388 + 19) in the post-CPOE period (Table 4). The implementation of CPOE was associated with a significant 17.5% reduction in the administration error rate (17.1% pre-CPOE versus 14.1% post-CPOE (p < 0.05)). Before and after CPOE implementation, more than half of the administration errors were administration omissions and CPOE had no impact on omissions. Interestingly, the administration of unordered drugs or of incorrect dosages showed a 2.5-fold decrease after the introduction of electronic prescribing (respectively 5.0% pre-CPOE versus 1.8% post-CPOE and 1.3% pre-CPOE versus 0.5% post-CPOE). However, we found a 65% increase in drugs administered at the incorrect time (0.7% pre-CPOE versus 2.0% post-CPOE, and these were mainly antithrombotic drugs (i.e. enoxaparin)). In the pre-CPOE period, we observed four administrations to the wrong patient, these errors were intercepted by the observer before reaching the patient (the nurse took the wrong patient pill dispenser). Administration to the wrong patient was not observed after the implementation of CPOE.

**Table 4:** Administration errors before and after CPOE implementation.

Type of error	Pre-CPOE (N = 1222)*	Post-CPOE (N = 1413)*	p Value
**Total (n, %)**	209 (17.1)	200 (14.1)	< 0.05
**Omission (n, %)**	120 (9.8)	140 (9.9)	
**Unordered drug (n, %)**	61 (5.0)	25 (1.8)	
**Incorrect dosage (n, %)**	16 (1.3)	7 (0.5)	
**Incorrect time (n, %)**	8 (0.7)	28 (2.0)	
**Wrong patient (n, %)**	4 (0.3)	0 (0)	

*Number of total opportunities for errors

## Discussion

CPOE implementation significantly decreased prescribing errors and administration errors and had no significant impact on errors at the dispensing stage.

Our study has several limitations. First, this single-center observational study with a before and after design has inherent associated drawbacks (e.g. limited external validity because of the single-center design, no randomization and no control group because of our observational non-controlled before-after design). In particular, such studies have a potential imbalance of unmeasured factors that might affect the differences in the two periods compared. In addition, the relatively short time-frames considered favor the risk of such an imbalance. Nevertheless, the study objective was not to demonstrate that CPOE results in a decrease in medication errors, taking into account any confounding factor. The objective of the study was to report a straightforward experience of the changes observed in medication errors, in a single department of orthopaedics switching from written orders to a computerized ordering system. In that regard, studying nearly 3000 medications prescribed to about 200 patients is amply sufficient for providing a reasonable global picture of the changes, a kind of snapshot. Another limitation of the study concerns the method of observation that may have changed the attitude of nurses in the dispensing and administration of drugs. However, Allan and Barker showed that the disguised observation decreases the Hawthorne effect on people observed.[28] In our study, the timing for evaluating the impact of electronic prescribing was 4 and 6 weeks after CPOE implementation, and the type and proportion of errors may evolve over time: in a recent study by Shulman et al [29], the error rate was monitored 2, 10, 25 and 37 weeks after CPOE implementation. Apart from an isolated increase at 10 weeks, the rate decreased over time. In our study, the impact of CPOE in time spent for prescribing, dispensing and administering drugs was not investigated. However, in a recent study, CPOE implementation was not associated with an increase in the time spent by doctors and nurses on direct patient care or medication-related tasks.[30] It is important to note that prescriptions were routinely checked by a pharmacist after CPOE implementation which might have influenced the results of our observation. In this complex intervention, our design did not allow us to distinguish the impact of CPOE versus the impact of a pharmacist. We were not able to calculate interobserver reliability as there was no cross observation. Finally, the impact of CPOE implementation on clinical outcomes was not explored in this study and only some types of errors were investigated (e.g. contraindication, drug-drug interactions, prescription of drugs despite an allergy were not studied). These issues should deserve future investigations.

Inherent to the computer system, the total eradication of procedural events with the CPOE is obvious and does not deserve further comments. The implementation of CPOE induced a dramatic 92% decrease in prescribing errors, close to that described by Reckmann et al. in a recent review.[31] The criteria chosen to evaluate the impact of CPOE on the prescription (clear prescription, route of administration, dosage and duplicated therapy) are simple criteria that determine, to a large extent, the quality of a medical prescription. For instance, illegibility or abbreviations are important issues as poor hand writing can induce harm to patients.[32, 33] Similar to our study, Westbrook et al showed that CPOE was efficient in decreasing administration route errors or dosage errors and Evans et al. showed elimination of prescriptions in which the route of administration or dosage were missing.[13, 15] These types of errors do not seem to be related to substantial negative outcomes for patients, however, they are time-consuming for nurses and their correction by CPOE is therefore relevant.[34] When a physician prescribes a medication with CPOE, some items must indeed be specified so that the doctor can validate prescription. This explains why the use of CPOE provides 100% of prescriptions with a dosage and the administration route.

Furthermore, CPOE allows for the creation of protocols including multiple drugs. These protocols do indeed enable faster and more comprehensive prescribing, but there are drawbacks related to the input of duplicated therapies. In our study, CPOE resulted in prescriptions of duplicated therapies, mainly related to drug orders overlapping in time. After surgery, anesthetists prescribe an analgesic protocol intravenously for the first 24 hours and an analgesic oral protocol for the following days. However, doctors may sometimes omit the important 24-hour time lag before the onset of the oral protocol thus starting their prescription at the same time as the intravenous protocol. Fortunately, these duplicated prescriptions did not lead to duplicated administration, as they were corrected by the nurse. Such errors of duplicated therapy orders were described in other studies on the impact of CPOE.[13, 14] In our study, the use of a CPOE system was associated with an increase in duplicated therapy (7/1593 (0.4%) vs 25/1388 (1.8%)). Physicians should however be aware of duplicated therapies as our CPOE system alerts them about these duplicated orders. However, the large number of alert messages sent by CPOE systems may overburden the attention of physicians.[35]

CPOE implementation had no significant impact on drug dispensing errors in our study. In our literature review, we found no studies evaluating the impact of CPOE on the dispensing of drugs by nurses from medicine trolleys. This might be due to the fact that in most American and British hospitals, drugs are dispensed on a daily basis to named patients and dispensing is carried out by the hospital pharmacy. In our study, the rate of dispensing errors was more than 30%, higher than that reported in other studies.[36, 37] Omission was the most frequent type of dispensing error. However and importantly, corresponding drugs were mainly analgesics and laxatives which were prescribed but administered only on patient demand. Therefore, nurses might not dispense drugs that they were not obliged to administer and would go to the satellite ward pharmacy to pick up the medication if needed.

In our study, CPOE implementation was associated with a 17.5% (17.1% vs 14.1%) decrease in medication administration errors. These results are in the range of administration error rates reported in a recent systematic review which was 10.5% [IQR: 7.3%-21.7%].[38] Studies assessing the rate of administration errors are numerous, but few assessed the impact of electronic prescribing on such errors. In a study by Bates et al,[24] the introduction of computerized prescribing resulted in a 59% reduction in administration errors, a rate much greater than that of our study. However, this may be explained by the fact that only correctly prescribed drugs were included in the evaluation of administration errors. We indeed stated that the quality of administration of a drug can not be assessed if it is not correctly prescribed.

Omission was the most common type of administration error observed. In about two-thirds of the cases (67.5%) the drugs involved in these omissions were analgesics that can be prescribed on a pro re nata basis. As nurses may adapt administration to patients’ pain, regardless of the prescription (on-demand or not), it seems difficult to assert that these omissions are potentially harmful errors for the patient or if they prevent drug overuse.

The administration of unordered drugs and incorrect dosages are the second most frequent errors after omissions. They have already been reported in the literature.[39, 40] In our study, implementation of CPOE resulted in a 3-fold decrease in these errors. CPOE provides a clear and precise prescription for nurses which may explain the decrease in the administration of incorrect dosage.

Before implementation of CPOE, incorrect administration time errors accounted for 3.8% (8/209) of administration errors whereas, in the literature this point is often the main cause of administration error.[9, 10] In our study, administration time in hand-written prescriptions was documented less than in computerized prescriptions (i.e. drugs were prescribed on a qd, bid or tid basis rather than with a precise schedule). As a result, only a limited number of incorrect administrations time could be detected in the pre-CPOE period. The fact that CPOE enables prescribers to label medication with a specific schedule may explain why the number of administrations at the incorrect time increased in the post-CPOE period (14% of administration errors). Therefore, since the procedures for documenting time of administration in handwritten and computerized orders are different, the differences in the observed rates of errors in time administration before and after CPOE implementation are hardly interpretable and should be considered with caution.

## Conclusion

The implementation of a CPOE in an orthopaedic surgery unit was associated with a dramatic global decrease in medication errors. Interestingly, CPOE was also associated with a minor number of errors related to surgery-specific patterns of prescription (i.e. duplicated orders and scheduled prescriptions not adapted to the nurse’s drug round after the 24-hour period following surgery), indicating that particular attention should be paid to the potential different prescriptions in the initial post-theater period.

## Supporting Information

S1 Dataset Individual data about patients’ characteristics and prescriptions used in the study. (XLS)

S1 Table Coding table for dataset. (DOC)
