# Supplementary material for: An Observational Study of the Impact of a Computerized Physician Order Entry System on the Rate of Medication Errors in an Orthopaedic Surgery Unit
Source: PLoS One. 2015 Jul 24;10(7):e0134101. doi: 10.1371/journal.pone.0134101 (PMC4514799; doi:10.1371/journal.pone.0134101)
Supplement: S1 Table — (DOC) [file pone.0134101.s002.doc]

S1 Table. Supporting information: Coding table

|  | Variable | Coding |
| --- | --- | --- |
| Patient information | Patient number | Incremental encoding |
|  | Gender | 0 M 1 F |
|  | Age (y) | Age |
|  | Length of hospitalization (d) | Length of hospitalization |
|  | Weight (kg) | Weight or ND: not determined |
|  | Type of hospitalization | 0 scheduled  1 unscheduled |
| Prescription | Type of prescription | 1 pre operative prescription  2 post operative prescription by the anaesthetist  3 post operative prescription by the surgeon  4 Personal patient’s prescription  NA non applicable |
|  | Prescriber | 1 senior anaesthetist  2 resident anaesthetist  3 senior surgeon  4 resident surgeon  ND non determined  NA non applicable |
|  | Legibility | 0 no 1 yes NA non applicable |
|  | Date present | 0 no 1 yes NA non applicable |
|  | Name of prescriber present | 0 no 1 yes NA non applicable |
|  | Signature of prescriber present | 0 no 1 yes NA non applicable |
|  | Date of birth present | 0 no 1 yes NA non applicable |
|  | Gender present | 0 no 1 yes NA non applicable |
|  | Posology present | 0 no 1 yes NA non applicable |
|  | Dosage present | 0 no 1 yes NA non applicable |
|  | Drug form present | 0 no 1 yes NA non applicable |
|  | Frequency present | 0 no 1 yes NA non applicable |
|  | Route of administration present | 0 no 1 yes 2 no route specified but only one route commercially available NA non applicable |
|  | Time of administration present | 0 no 1 yes NA non applicable |
|  | PRN | 0 no 1 yes NA non applicable |
| Information on the drug | Name of drug | Brand name |
| Drug information at the stage of dispensing | Form of drug dispensed | 1 tablet, capsule  2 gum  3 oral solution  4 cutaneous  5 patch  6 ophthalmic  7 otic  8 nasal  9 suppository  10 ovule  11 Inhaler  12 Intravenous, intramuscular, subcutaneous  NA non available |
|  | Quantity dispensed | Number of tablets, capsules… |
|  | Drug in the patient’s rack | 0 no 1 yes |
| Drug information at the stage of administration | Quantity administered | Number of tablets, capsules… |
|  | Drug administered | 0 no 1 yes NA non applicable |
|  | Route of drug administered | 1 oral  2 IV  3 S/C  4 IM  5 cutaneous  6 rectal  7 inhalation  8 ophthalmic  9 otic  10 sublingual  11 other  NA non applicable |
|  | Reason of non administration | 1 patient missing or sleeping / 2 wait meal / 3 vomiting / 4 no vein available/ 5 on demand / 6 patient refusal / 7 substitution / 8 patient discharged / 9 at bedtime / 10 wait for blood test / 11 later administration / 12 not prescribed at this time / 13 unordered / NA non applicable / ND non determined |
| Prescribing error | Wrong form prescribed | 0 no 1 yes NA non applicable |
|  | Wrong posology prescribed | 0 no 1 yes NA non applicable |
|  | Wrong time prescribed | 0 no 1 yes NA non applicable |
| Dispensing error | Wrong dosage dispensed | 0 no 1 yes NA non applicable |
|  | Wrong form dispensed | 0 no 1 yes NA non applicable |
|  | Wrong posology dispensed | 0 no 1 yes NA non applicable |
|  | Wrong time dispensed | 0 no 1 yes NA non applicable |
| Administration error | Wrong dosage administered | 0 no 1 yes NA non applicable |
|  | Wrong form administered | 0 no 1 yes NA non applicable |
|  | Wrong posology administered | 0 no 1 yes NA non applicable |
|  | Wrong time administered | 0 no 1 yes NA non applicable |
| Unordered drug | Unordered drug dispensed and/or administered | 0 no 1 yes NA non applicable |
